# Cardiac Dysfunction in the BACHD Mouse Model of Huntington’s Disease

**DOI:** 10.1371/journal.pone.0147269

**Published:** 2016-01-25

**Authors:** Analyne M. Schroeder, Huei Bin Wang, Saemi Park, Maria C. Jordan, Fuying Gao, Giovanni Coppola, Michael C. Fishbein, Kenneth P. Roos, Cristina A. Ghiani, Christopher S. Colwell

**Affiliations:** 1 Department of Psychiatry & Biobehavioral Sciences, University of California Los Angeles, Los Angeles, California, 90095–1751, United States of America; 2 Department of Physiology and Cardiovascular Research Lab, University of California Los Angeles, Los Angeles, California, 90095–1751, United States of America; 3 Department of Pathology & Laboratory Medicine, University of California Los Angeles, Los Angeles, California, 90095–1751, United States of America; Rutgers New Jersey Medical School, UNITED STATES

## Abstract

While Huntington’s disease (HD) is classified as a neurological disorder, HD patients exhibit a high incidence of cardiovascular events leading to heart failure and death. In this study, we sought to better understand the cardiovascular phenotype of HD using the BACHD mouse model. The age-related decline in cardiovascular function was assessed by echocardiograms, electrocardiograms, histological and microarray analysis. We found that structural and functional differences between WT and BACHD hearts start at 3 months of age and continue throughout life. The aged BACHD mice develop cardiac fibrosis and ultimately apoptosis. The BACHD mice exhibited adaptive physiological changes to chronic isoproterenol treatment; however, the medication exacerbated fibrotic lesions in the heart. Gene expression analysis indicated a strong tilt toward apoptosis in the young mutant heart as well as changes in genes involved in cellular metabolism and proliferation. With age, the number of genes with altered expression increased with the large changes occurring in the cardiovascular disease, cellular metabolism, and cellular transport clusters. The BACHD model of HD exhibits a number of changes in cardiovascular function that start early in the disease progress and may provide an explanation for the higher cardiovascular risk in HD.

## Introduction

Over the course of a lifetime, Huntington’s disease (HD) patients are subject to a progressive neurodegenerative process that inflicts cognitive, psychiatric and motor dysfunction [1, 2]. HD is caused by a CAG repeat expansion within the first exon of the *huntingtin* (*Htt*) gene and when translated, produces a polyglutamine (polyQ) repeat that leads to protein misfolding, soluble aggregates, and inclusion bodies detected throughout the body [3, 4]. The normal function of the protein (HTT) is unknown; however, the mutated form leads to dysfunction of a large range of cellular processes including cytoskeletal organization, protein folding, metabolism and transcriptional activities. Cardiovascular events are a major cause of early death in the HD population and these events occur at higher rates compared to the rest of the population [5–7]. The limited cardiovascular studies in HD patients attribute the increased cardiovascular susceptibility in part to a dysfunctional autonomic nervous system (ANS) that can be detected early in the disease progression [8–13]. Additional studies are needed to better understand the time-course and cause of cardiovascular pathology in HD that could potentially help improve symptoms and prevent early death.

While there is no perfect animal model of HD [14], several models recapitulate aspects of the cardiovascular dysfunction seen in human disease. For example, dysfunction of the ANS has been reported in the several mouse models of HD as measured by heart rate variability and baroreceptor reflex experiments [15–18]. Reduced contractility and cardiac output is also a common feature in mouse models [15–16,19–21]. The cardiac-specific expression of polyQ repeats leads to dysfunction, suggesting that cardiovascular disease is a result of both local dysfunction as well as improper input [22, 23]. Advances in these preclinical models are critical if we are to develop a mechanistic understanding of the cardiovascular pathology in HD but establish models to evaluate therapeutic interventions.

In this study, we sought to define the time course of cardiovascular symptoms in HD using the BACHD mouse model: a bacterial artificial chromosome (BAC) mediated transgenic whereby the mutant form of the full length human *Htt* gene with 97 stable CAG repeats was incorporated into the mouse genome [24]. Using this disease model, we evaluated the age-dependent progression in heart dysfunction using echocardiograms starting at the beginning of motor symptoms at 3 mo of age and progressing to an age (15 mo) when the motor symptoms are pronounced and brain atrophy can be detected. In addition, we assessed the BACHD heart’s response to chronic treatment with beta-adrenergic receptor agonist isoproterenol (ISO). Finally, we examined gene expression profiles in the heart early (3 mo) and late (15 mo) in the disease progression in the BACHD model.

## Materials and Methods

### Experimental Animals and Ethics Statement

BACHD mice on the C57BL6/J background along with littermate wild-type (WT) controls were acquired from the mouse mutant resource at The Jackson Laboratory, (JAX, Bar Harbor, Maine) in a colony maintained by the CHDI Foundation. The mice start showing symptoms at 2–3 mo of age and by 12 mo manifest full symptoms. In our colony, the BACHD mice commonly live to 18 mo of age but their health declines precipitously after 16 mo. Therefore, we stopped the present study when mice reached 15 mo of age. Five separate cohorts of mice of each genotype were used for this study: 1) longitudinal measurement of cardiac function using echocardiograms; 2) chronic treatment with isoproterenol (ISO) to boast cardiac output from 3 to 6 mo of age; 3) transcriptional comparison between the hearts of genotypes at 3 and 15 mo of age; 4) measurement of serum cytokines in 3 mo old animals using a multiplex assay; 5) measurement of apoptotic marker in 12–15 mo hearts by western blot. All procedures followed guidelines of the National Institutes of Health and were approved by the UCLA Animal Research Committee.

To provide some details, the health of the mice was monitored daily. We looked for a set of symptoms including restlessness, impaired mobility, licking or guarding wound, failure to groom, open sores, loss of appetite or weight loss. At any sign of ill health, a member of the veterinary staff was consulted for course of treatment. If the animal did not improve, then it would be humanely sacrificed with an overdose of isoflurane and followed by decapitation. For pain management, the mice were given carprofen at 5 mg/kg every 24 hrs for 48 hr minimum. The first dose was given before surgery, and the remaining post-operatively.

### Long-Term Assessment of the Heart in WT and BACHD Mice

BACHD mice (*n* = 10) and WT (*n* = 10) were group housed and kept in a 12:12 light/dark (LD) cycle with rodent chow provided ad libitum. Mice were examined with echocardiograms at 3, 6, 9, 12 and 15 mo of age. After the last echocardiogram measure, the mice were perfused and morphologic and histological measurements were taken. During the course of the study, 1 BACHD and 2 WT mice died and the data from these mice were excluded from the analysis.

### Echocardiograms

Echocardiograms were measured using a Siemens Acuson Sequoia C256 instrument equipped with a 15L8 15MHz probe (Siemens Medical Solutions, Mountain View, CA) as previously described [16]. Briefly, two-dimensional, M-mode echocardiography and spectral Doppler images enabled measurement of heart dimension and function (Left Ventricle (Lv) Mass), end-diastolic dimension (EDD), end-systolic dimension (ESD), posterior wall thickness (PWT), ventricular septal thickness (VST), Lv Ejection Fraction (Lv EF). The mice were sedated with 1% isoflurane vaporized in oxygen (Summit Anesthesia Solutions, Bend, OR) and HR was monitored using *electrocardiogram* to maintain physiological levels (between 450 and 650).

### Isoproterenol (ISO) Treatment

BACHD and WT mice (2–3 mo) were subjected to echocardiograms before subcutaneous implantation of an osmotic pump (Alzet model 2001, Durect Corp, Cupertino, CA) containing either saline (WT: *n* = 4; BACHD: *n* = 4) or ISO (WT: *n* = 10; BACHD: *n* = 8). To determine an effective dose of isoproterenol, each treated mouse received sequentially increased doses of ISO, beginning with 0.24 mg/day, then 0.48 mg/day, and finally 0.97mg/day. It was this final concentration that proved effective at significantly increasing HR and was used in this study. At the end of the treatments (3 months duration), cardiovascular function was assessed using electrocardiogram recording and echocardiography. After the last echocardiogram measure, the mice were perfused and morphologic and histological measurements were taken.

### Electrocardiogram (ECG) Measurements

ECG traces were obtained under isoflurane anesthesia by inserting two platinum needle electrodes (Grass Technologies, West Warwick, RI) under the skin in the lead II configuration. The ECG signal was amplified (Grass Technologies), acquired and analyzed with HEM V4.2 software (Notocord Systems, Croissy sur Seine, France). The ECG data were recorded for 5 to 10 mins from each mouse monthly for the duration of the study. Heart rate (HR) was calculated using the RR-interval for all animals.

### Morphometry and Histology

Following the longitudinal echocardiogram measurements as well as the ISO treatments (described above), animals were deeply anaesthetized using isoflurane and perfused with phosphate-buffered saline (PBS, pH 7.4) with heparin (2 units/ml, Henry Schein, Melville, NY) followed by 4% (w/v) paraformaldehyde (Sigma-Aldrich) in PBS (pH 7.4). A motorized pump was used to deliver the solutions in order to control and maintain similar pressure between animals. Hearts were dissected, weighed and post-fixed at 4°C. Tibia length (TL) was also measured. Heart weight (HW)/TL and HW/body weight (BW) ratios were calculated to determine any differences in morphometry that would indicate cardiac hypertrophy. Following a series of dehydration steps using ethanol and xylene, hearts were embedded in paraffin. Paraffin-embedded hearts were sectioned and stained with H&E or Masson’s Trichrome. Heart dimensions were measured using two mid-ventricular cross sections from each H&E and/or Masson’s Trichrome-stained heart with the aid of Image J software. The presence or absence of fibrotic staining was visually scored by two researchers masked to the experimental conditions. In order to quantitate the degree of fibrosis, images were acquired from each heart. Areas of fibrosis were captured, and in sections lacking positive fibrotic staining, an area of the left ventricle (Lv) wall was imaged. Pictures were processed using Corel Draw, to remove pixels of background and pink cardiomyocyte tissue, leaving areas of positive blue stain in the image. Integrated density of these processed images was measured using Image J, where pixel areas less than 3 were excluded from the measurements in order to minimize noise. The resulting density numbers were divided by the area of the heart tissue in the image.

Trichrome stained sections were used to estimate the cardiomyocite cross-sectional area in aged (15 mo) WT and BACHD mice (n = 4 per group). Measurements were performed by three observers masked to the genotype of the mice. Images from multiple fields (3–5) at the level of the papillary muscles were acquired on a Zeiss Axioskop with an Axiocam using the AxioVision software (Zeiss, Pleas- anton, CA, USA), and measurements (in μm) obtained using the AxioVision software. Only cells with a well-defined round shape were considered. The cross-sectional areas of 7–12 cells/animal were averaged and analyzed for statistical difference.

### RNA Extraction

A separate cohort of young (3 mo) and older (15 mo) WT and BACHD mice (*n* = 4 per group) were used for gene expression analysis. Total RNA from the ventricles were extracted using Trizol RNA isolation protocol (LifeTechnologies, Carlsbad, CA), then DNAse treated (TURBO DNA-*free* kit, Life Technologies) and column purified (Pureline RNA Mini-kit, Life Technologies). RNA was used for microarray and Quantitative Real-Time PCR experiments described below.

### Microarray Hybridization

Microarray processing was performed by the Southern California Genotyping Consortium at UCLA. Briefly, whole genome gene expression profiling protocols began with 100 ng of total RNA isolated as described above. Samples were quantitated using Ribogreen fluorescent assay and normalized to10 ng/ul prior to amplification. Amplified and labeled cRNA was produced using the Illumina-specific Ambion TotalPrep kit. 1st and 2nd strand cDNA was produced using the Ambion kit (Life Technology) and purified using a robotic assisted magnetic capture step. Biotinylated cRNA was produced from the cDNA template in a reverse transcription reaction. After a second Ribogreen quant and normalization step, amplified and labeled cRNA was hybridized overnight at 58°C to Illumina MouseRef-8 v2.0 expression arrays. Hybridization was followed by washing, blocking, staining and drying on a Little Dipper processor. Array chips were scanned using an iScan reader, and expression data were extracted and compiled using BeadStudio software (Illumina, San Diego, CA).

### Quantitative Real-Time PCR

One μg of total RNA was reverse transcribed using High Capacity cDNA Reverse Transcription Kit (Applied Biosystems, Foster City, CA). Quantitative real-time RT-PCR was performed using SYBR Green Mix (Applied Biosystems, Foster City, CA). Using Primer3 [25] following primers were designed to flank intron-exon broundaries: *Hspa1a* (sense: 5’-GGT CTC AAG GGC AAG CTC AG-3’ and anti-sense: 5’-CTT GTG CAC GAA CTC CTC CT-3’) *Nppb* (sense: 5’-TCC TCT GGG AAG TCC TAG CC-3’ and anti-sense: 5’-AGC TGT CTC TGG GCC ATT TC-3’) *Kcnip2* (sense: 5’-AAT CCC GAG ATT TGG ACG GC-3’ and antisense: 5’-GCT TGA GGA AAC GCT GCT TC-3’) *Acot1* (sense: 5’-AGA AGC CGT GAA CTA CCT GC-3’ and antisense: 5’-GGA AGG AGG CCA TAG CAA GG-3’) *Tbp* (sense: 5’ CAC GGA CAA CTG CGT TGA TT-3’ and antisense: 5’-AGC CCA ACT TCT GCA CAA CT-3’) *B2M* (sense: 5’-GGT GAC CCT GGT CTT TCT GG-3’ and antisense: 5’-TGT TCG GCT TCC CAT TCT CC-3’). Specificity and efficiency of the reactions were verified using melting curves and dilution series, respectively. Expression levels of *Hspa1a*, *Nppb*, *Kcnip2*, and *Acot1* were calculated using the 2^-ΔΔCt^ method where *Tbp* and *B2m* were used as normalizing genes. *Tbp* and *B2m* did not vary between genotypes and we report gene expression normalized to *Tbp*.

### Multiplex Assay

Blood sample was collected from a separate cohort of young (3-4mo) WT (*n* = 8) and BACHD (*n* = 8) mice using the facial vein technique. Blood was allowed to clot for at least 30 mins at room temperature, and serum was collected following centrifugation at 3000rpm for 15 mins and frozen at -80^C^ until analyzed. A volume of 25μL serum was used to assess cytokine levels of IFNγ, IL-10, IL-12, IL-1A, IL-1B, IL-2, MIP1A, RANTES, IL-6, MCP-1, and TNF-α using the Millipore cytokine milliplex kit (EMD Millipore Corporation, Billerica, MA, USA) and the Biorad Bio-plex 200 systems machine (Hercules, CA, USA).

### Western Blot

Hearts from aged WT (n = 4) and BACHD (n = 4) mice were rapidly dissected and homogenized in lysis buffer containing 50 mM Tris/HCl, 0.25% (w/v) DOC (sodium deoxycholate), 150 mM NaCl, 1 mM EDTA, 1nM EGTA, 1% (w/v) Nonidet P40, 1 mM Na3VO4 (sodium orthovanadate), 1 mM AEBSF [4-(2-aminoethyl)benzenesulfonyl fluoride], 10 μg/ml aprotinin, 10 μg/ml leupeptin, 10 μg/ml pepstatin and 4 μM sodium fluoride. Total protein concentration in cleared extracts was estimated using the Thermo Scientific™ Pierce™ BCA Protein Assay Kit (Thermo Fisher Scientific, Waltham, MA USA). Fifty μg of total proteins were loaded on to a 4–20% Tris-glycine gel (Invitrogen, Carlsbad, CA). Western blots were performed as previously described [26]. Equal protein loading was verified by Ponceau S solution (Sigma, Saint Louis, MO) reversible staining of the blots. Each extract was analyzed for the relative protein levels of Heat-Shock protein (Hsp)-70, by using a mouse monoclonal antibody (Sigma, Saint Loius, Mo), and Cleaved Caspase-3, by using rabbit polyclonal antibodies (EMD Millipore Corporation, Billerica, MA & Cell Signaling, Danvers, MA). Each extract was also analyzed for relative protein levels of GAPDH (Genetex, Irivine, CA) by stripping and re-probing. Protein bands were detected by chemiluminescence using the Thermo Scientific™ Pierce™ ECL 2 Western Blotting Substrate or the Amersham ECL kit (GE Healthcare; Piscataway, NJ) with HRP (horseradish peroxidase)-conjugated secondary antibodies (Cell Signaling, Danvers, MA). Relative intensities of the protein bands were quantified by scanning densitometry using the NIH Image Software (Image J, http://rsb.info.nih.gov/ij/). For the comparison of relative protein levels in the heart from WT and BACHD animals, each background-corrected value was normalized according to the relative GAPDH levels of the sample, and then referred to the average of the WT values calculated from the same immunoblot image.

### Statistical Analysis

A two-way repeated measures ANOVA was used to analyze the long-term changes in echocardiographic measurements between WT and BACHD animals using genotype and age as factors. Other comparisons between the two genotypes were made using a Student’s *t*-test. If the data set failed to pass a test for equal variance, a Rank-Sum Test was used. These statistical analyses were performed using the SigmaStat (San Jose, CA) or Prism 5 (GraphPad Software, San Diego, CA) statistical software. Data are shown as the mean ± SEM.

Raw microarray data were analyzed by using Bioconductor packages as previously described [27]. Briefly, quality assessment was performed by examining the inter-array Pearson correlation and clustering based on the top variant genes. Contrast analysis of differential expression was performed by using the LIMMA (Linear Models for Microarray and RNA-Seq Data) package [28]. After linear model fitting, a Bayesian estimate of differential expression was calculated, and the *P*-value threshold was set at 0.005. The fold change of gene expressions is calculated by comparing the signal intensity of young hearts and old hearts. Differentially expressed genes were then classified according to gene ontology and pathways using Ingenuity Pathway Analysis (IPA, Ingenuity Systems, Redwood City, CA). After applying threshold at P = 0.005, the top 10 genes up-regulated and down-regulated in BACHD compared to WT at the same age are selected according to their P-value. In other words, they are the most significantly changed genes in the comparison.

## Results

### Structural and Functional Differences between WT and BACHD hearts Start at 3 mo of Age

**Fig 1:**
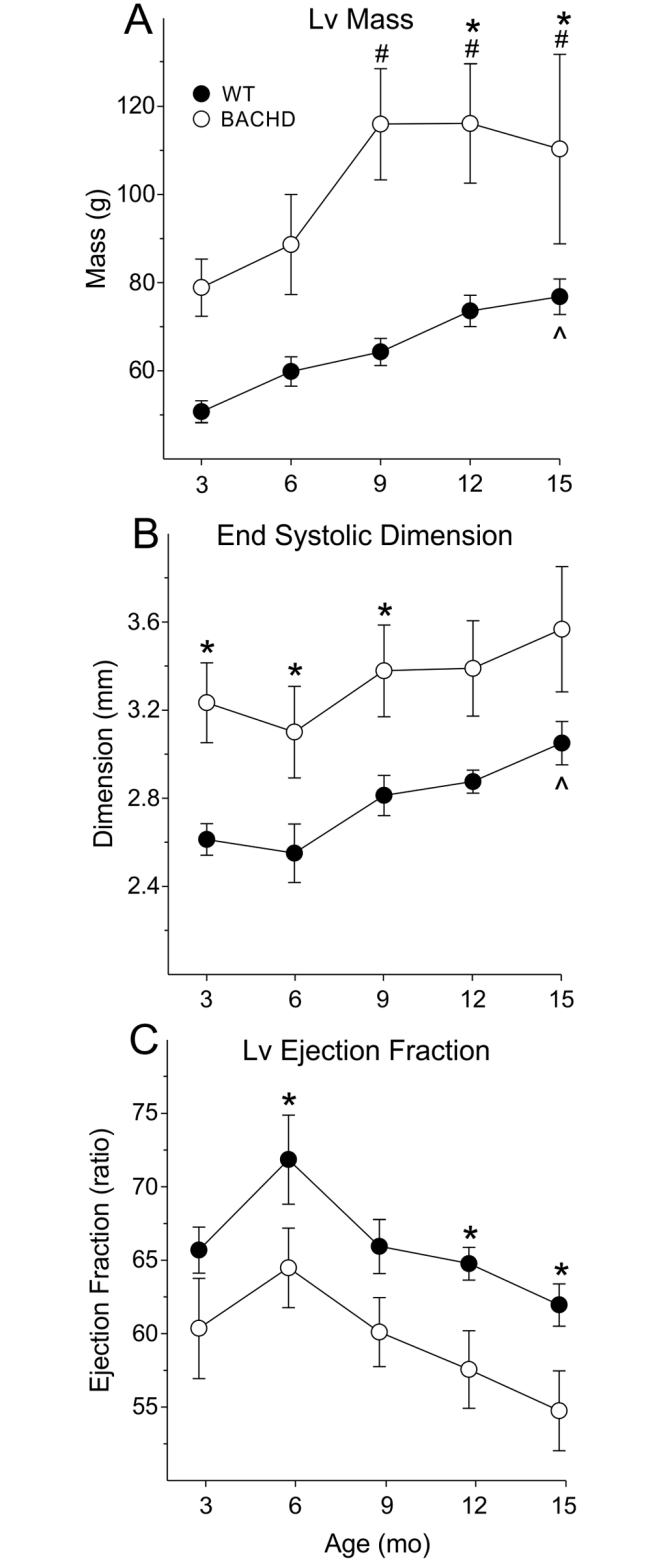
Echocardiogram indicates structural and functional differences early in the disease progression. Structural (A) and functional (B, C) parameters of the heart from WT (n = 8) and BACHD (n = 9) mice were measured from 3 to 15 mo of age. ^ *P*<0.05 within WT vs. 3 mo. ^#^ *P*<0.05 within BACHD vs. 3 mo. * *P*<0.05 for genotypic differences.

We assessed cardiac structure and function in a group of BACHD and WT mice throughout most of their adult life in order to follow changes in cardiovascular parameters and establish a time course for pathology. We use echocardiography to follow the same BACHD and WT littermate control mice longitudinally from 3 to 15 mo (Fig 1; Table 1). The hearts of WT mice slowly increased in dimension as they aged, with significance in EDD and PWT starting at 12 mo while Lv Mass and ESD were significantly larger at 15mo (Fig 1A and 1B; Table 1). Cardiac function as measured by Lv EF showed an increase between 3 and 6 mo as is typical with development of WT mice and then declined with age (Fig 1C). The hearts of BACHD mice also increased in size as they aged, with a significant increase in Lv Mass and PWT measures starting at 9 mo of age (Fig 1A; Table 1). Cardiac function of BACHD mice did not significantly change with age (Fig 1C; Table 1). The hearts of BACHD mice were larger (ESD and EDD) and thicker (PWT) than WT mice starting at 3 mo of age (Fig 1B; Table 1). Lv Mass was significantly higher in BACHD compared to WT with significant differences starting at 9 mo (Fig 1A). Functional measurements (Lv EF) were depressed in BACHD mice compared to WTs starting at 3 mo of age, and remained lower at all ages (Fig 1C). Throughout adult life, the hearts of BACHD mice were larger and functionally depressed compared to WTs.

**Table 1:** Echocardiographic parameters in BACHD and WT animals beginning at 3 mo of age.

	WT (*n* = 8)					BACHD (*n* = 9)					2-way ANOVA		
Age (mo)	3	6	9	12	15	3	6	9	12	15	*Genotype*	*Age*	*Interaction*
**Lv Mass (g)**	51±2	60±3	64±3	74±4	77±4*	79±6	89±11	116±13†‡	116±13†‡	110±21†‡	*F*_*1*_ = 7.5, *P* = 0.02	*F*_*4*_ = 11.5,*P<* 0.001	*F*_*4*_ = 1.64,*P* = 0.18
**ESD (mm)**	2.6±0.1	2.5±0.1	2.8±0.1	2.9±0.1	3.0±0.1*	3.2±0.2‡	3.1±0.2‡	3.4±0.2‡	3.4±0.2	3.6±0.3	*F*_*1*_ = 6.2, *P* = 0.03	*F*_*4*_ = 7.8, *P<* 0.001	*F*_*4*_ = 0.11, *P* = 0.98
**EDD (mm)**	3.8±0.1	3.9±0.1	4.1±0.1	4.2±01*	4.4±0.1*	4.5±0.1‡	4.4±0.2	4.6±0.2‡	4.6±0.2	4.7±0.3	*F*_*1*_ = 4.7,*P* = 0.046	*F*_*4*_ = 6.2,*P<* 0.001	*F*_*4*_ = 1.21,*P* = 0.32
**PWT (mm)**	0.44±0.02	0.48±0.01	0.48±0.01	0.52±0.01*	0.50±0.02	0.50±0.01‡	0.55±0.02‡	0.64±0.02†‡	0.66±0.02†‡	0.58±0.03†‡	*F*_*1*_ = 36.5, *P<* 0.001	*F*_*4*_ = 15.0, *P<* 0.001	*F*_*4*_ = 3.08,*P* = 0.02
**LvEF (ratio)**	66±2	72±3	66±2	65±1	62±1	60±3	64±3‡	60±2	57±2‡	54±3‡	*F*_*1*_ = 6.1,*P* = 0.030	*F*_*4*_ = 8.2,*P<* 0.001	*F*_*4*_ = 0.14,*P* = 0.97

* *P*<0.05 within WT, significant difference vs 3 mo;

^†^ *P*<0.05 within HD, significant difference vs 3 mo;

^‡^ *P*<0.05 significant difference between genotypes in the same age group.

### Aged BACHD Mice Develop Cardiac Fibrosis

**Fig 2:**
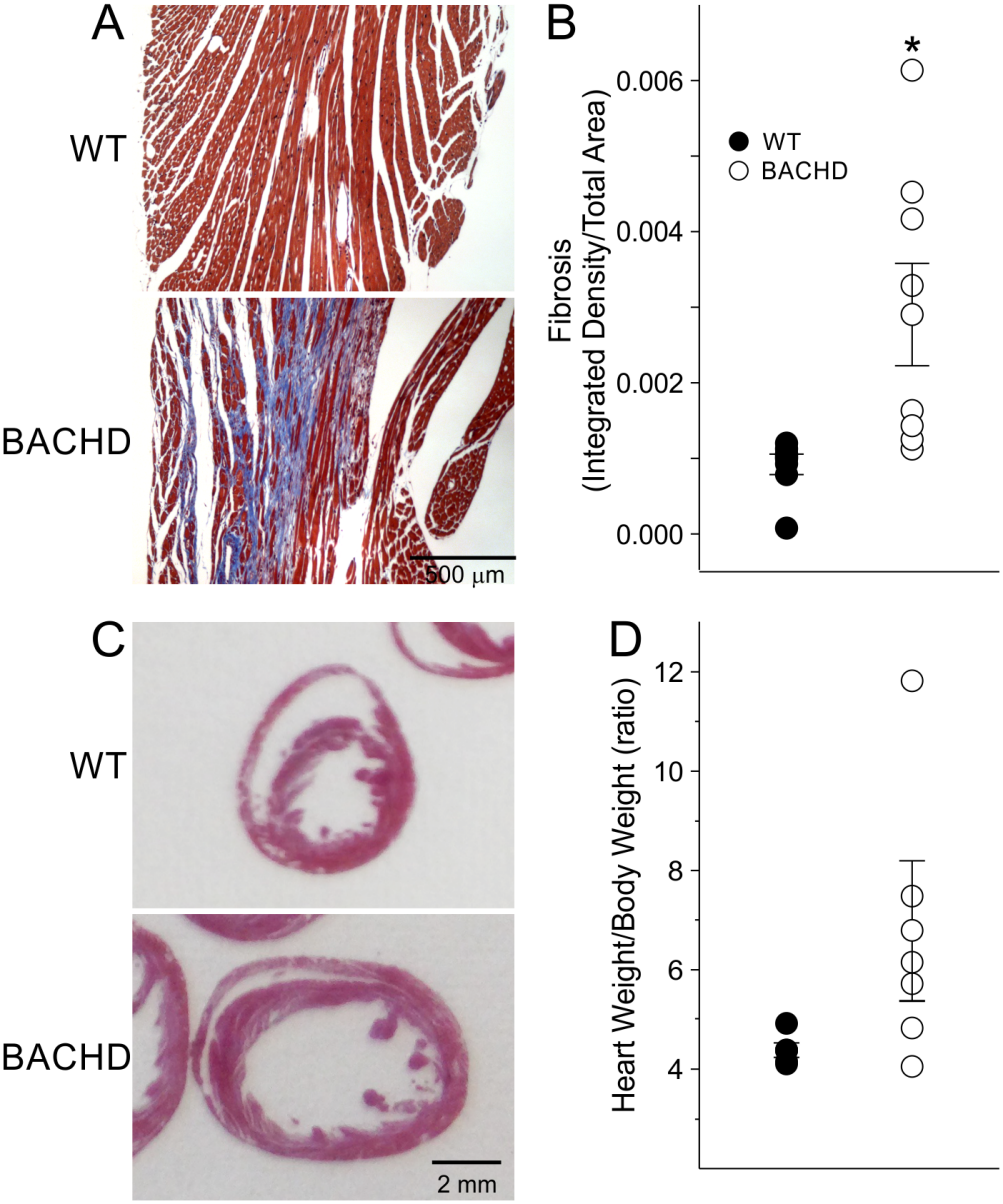
Histological analysis finds evidence of fibrosis and hypertrophy in the BACHD hearts. Examples of Masson’s Trichrome stained heart sections from 15 mo old WT and BACHD mice (A). Quantification of fibrosis by measuring the integrated density of fibrotic tissue and divided by tissue area detected significantly increased areas of fibrosis in BACHD mice (B). Examples of hematoxylin and eosin (H&E) stained heart sections from 15 mo old WT and BACHD mice (C). Morphometry measurements comparing heart weight relative to body weight between 15 mo old WT and BACHD mice (D). * *P*<0.05 for genotypic differences.

To further assess the cardiac structure of WT and BACHD hearts, we sacrificed the mice at about 15 mo of age and processed their hearts Masson’s Trichrome to visualize fibrotic tissue or H&E staining. We did not detect any fibrotic staining in the WT hearts, while positive staining was present in 6 of the 9 BACHD mice (Fig 2A). Optical density measurements of fibrotic staining were significantly higher in BACHD mice compared to WTs (Fig 2B; Table 2). BACHD hearts displayed enlarged Lv (Fig 2C). Although these values did not reach significance, an increase in Lv diameter (19%) and septal thickness (20%) were observed in BACHD compared to WT, whilst no difference in right ventricle dimensions were found between genotypes (data not shown). Furthermore, no changes were found in the cross-sectional area of the BACHD cardiomyocytes (103 ± 15 μm^2^; n = 4) as compared to WT (121 ± 13 μm^2^, n = 4).

**Table 2:** Histological measurements of hearts of 15 mo WT and BACHD mice.

Histological Measurements	WT (*n* = 8)	BACHD (*n* = 9)	*t*-test
**Heart Weight/Body Weight**	4.4 ± 0.1	6.6 ± 2.8	*T* = 1.72, *P* = 0.119
**Heart Weight/Tibia Length**	10.0 ± 0.1	12.8 ± 2.1	*T* = 21.0, *P* = 0.126
**Heart Weight (mg)**	180 ± 2	220 ± 33	*T* = 21.5, *P* = 0.126
**Body Weight (g)**	41 ± 2	35 ± 4	*T* = 1.43, *P* = 0.187
**Tibia Length (mm)**	18.0 ± 0.1	18.0 ± 0.1	*T* = 0.31, *P* = 0.761
**Fibrosis (integrated density/total area)**	9.2 ± 1.4	29.0 ± 6.8*	*T* = 45.0, *P* = 0.015

* *P*<0.05 significant difference between genotypes.

In agreement with life-long abnormalities in the BACHD hearts, the morphometric measurements (HW, HW/TL and HW/BW ratios) were all larger in the BACHD mice, although these differences were not significant due to the high variability in the mutant mice (Fig 2D; Table 2). In summary, at 15 mo of age, morphometric analysis showed a trend toward hypertrophy and a significant increase in fibrotic lesions in the hearts of BACHD mice.

### Hearts of BACHD Mice Displayed Structural and Functional Adaptation to Chronic β-Adrenergic Treatment

**Fig 3:**
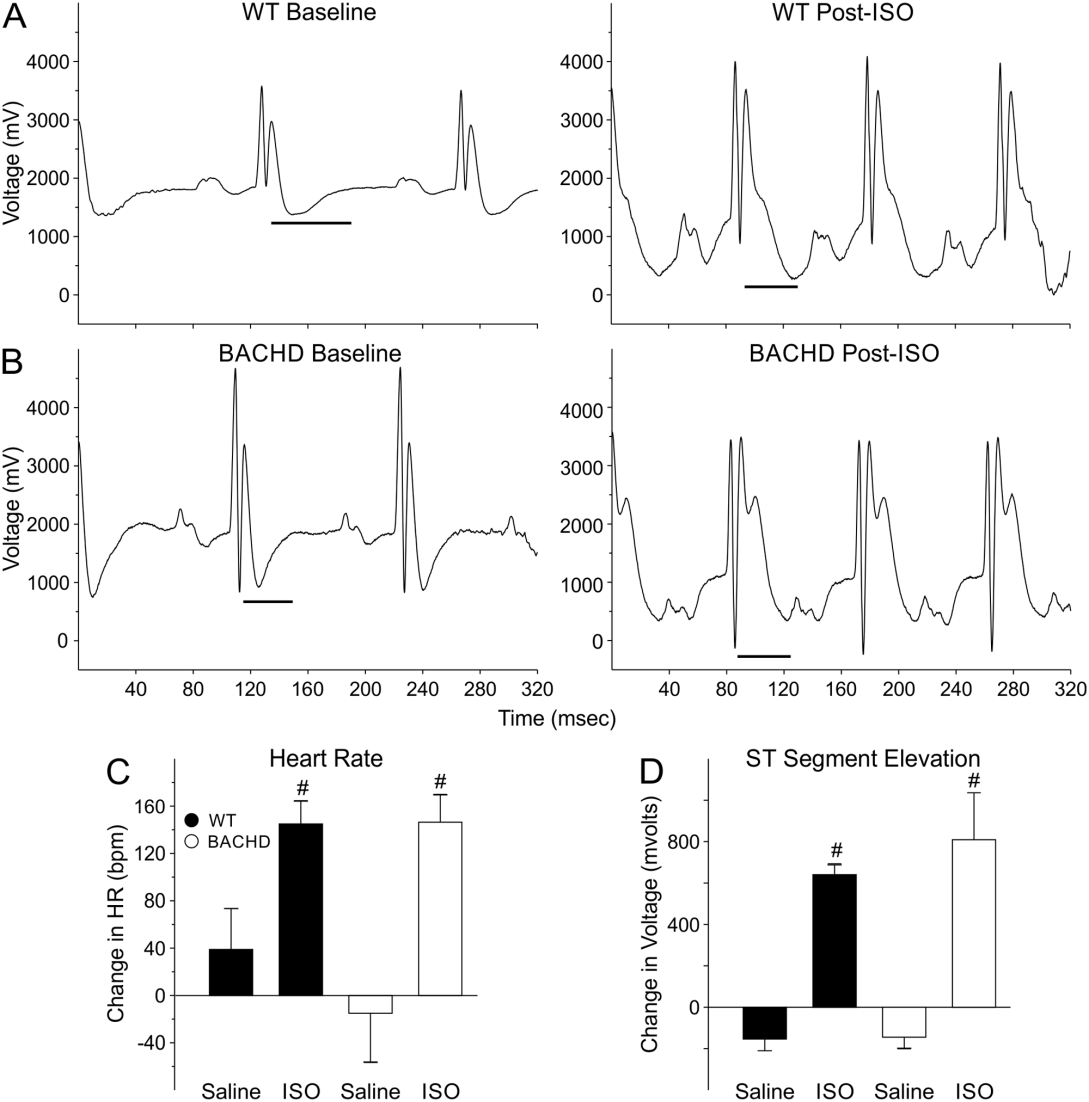
The cardiovascular system of the BACHD mice responded adaptively to chronic treatment with an adrenoreceptor challenge (ISO, 0.96mg/kg per day for 30 days). Examples of ECG waveforms from WT (n = 10) (A) and BACHD (n = 8) mice (B). The ST segment range is shown by the black line. ISO increased HR (C) and elevated the ST segment voltage (D) in both genotypes indicating that the drug was effective in stimulating the heart also in BACHD mice. No significant difference in the response to ISO between genotypes was detected. ^#^ *P*<0.05 vs. saline.

In previous work, we have shown that the BACHD mice show an abnormally weak baroreceptors response to decreases in blood pressure, consistent with a weak sympathetic regulation of heart rate [16]. Therefore, we chronically treated 3mo BACHD and WT mice with ISO to see if there were genotypic differences in the response to this stimulant. By activating beta-adrenergic receptors in the heart, ISO increases heart rate and mimics the action of the sympathetic nervous system. Before drug treatment, there was no difference in baseline HR (WT: 457 ± 21 bpm; BACHD: 498 ± 16 bpm). ISO increased HR of both WT (612 ± 11 bpm) and BACHD (621 ± 16 bpm) animals indicating that it was having the desired effect of stimulating β-adrenergic pathways (Fig 3A and 3B). Saline treatment did not alter the HR in either genotype (Fig 3C). The magnitude changes in HR post-treatment were not significantly different between WT and BACHD mice suggesting similar drug responsiveness between genotypes (Fig 3C). The ST-segments of the waveforms were elevated in ISO-treated WT and BACHD mice suggesting that the hearts were under ischemic conditions as a result of the cardiovascular challenge. The Lv EF was significantly reduced in the BACHD animals compared to WT controls (Table 3). In summary, ECG measurements suggest that both WT and BACHD mice responded to ISO treatment as measured by increases in HR and ST-segment voltages. The Lv EF was reduced in the mutants, but otherwise, the echocardiograms did not find significant differences in the response to the ISO treatment between the genotypes. These results suggest that BACHD mice are adapting to the cardiovascular challenge and that the beta-adrenergic receptors are functioning properly.

**Table 3:** Echocardiographic parameters from BACHD and WT animals (6 mo) following chronic administration of isoproterenol (0.97 mg/day for 30 days).

Echo Measurements	WT (*n* = 10)	BACHD (*n* = 8)	*t*-test
**Lv Mass (mg)**	73 ± 3	94 ± 11	*T* = -1.8, *P* = 0.08
**ESD (mm)**	2.9 ± 0.1	3.4 ± 0.3	*T* = -1.9, *P* = 0.06
**EDD (mm)**	4.2 ± 0.1	4.4 ± 0.3	*T* = -0.7, *P* = 0.45
**PWT (mm)**	0.51 ± 0.01	0.55 ± 0.02	*T* = -1.5, *P* = 0.14
**Lv EF**	67 ± 3	56 ± 3*	*T* = 2.59, *P* = 0.01

* *P*<0.05 significant difference between genotypes.

### Chronic β-Adrenergic Treatment Exacerbated Fibrotic Lesions in the Hearts of BACHD Mice

**Fig 4:**
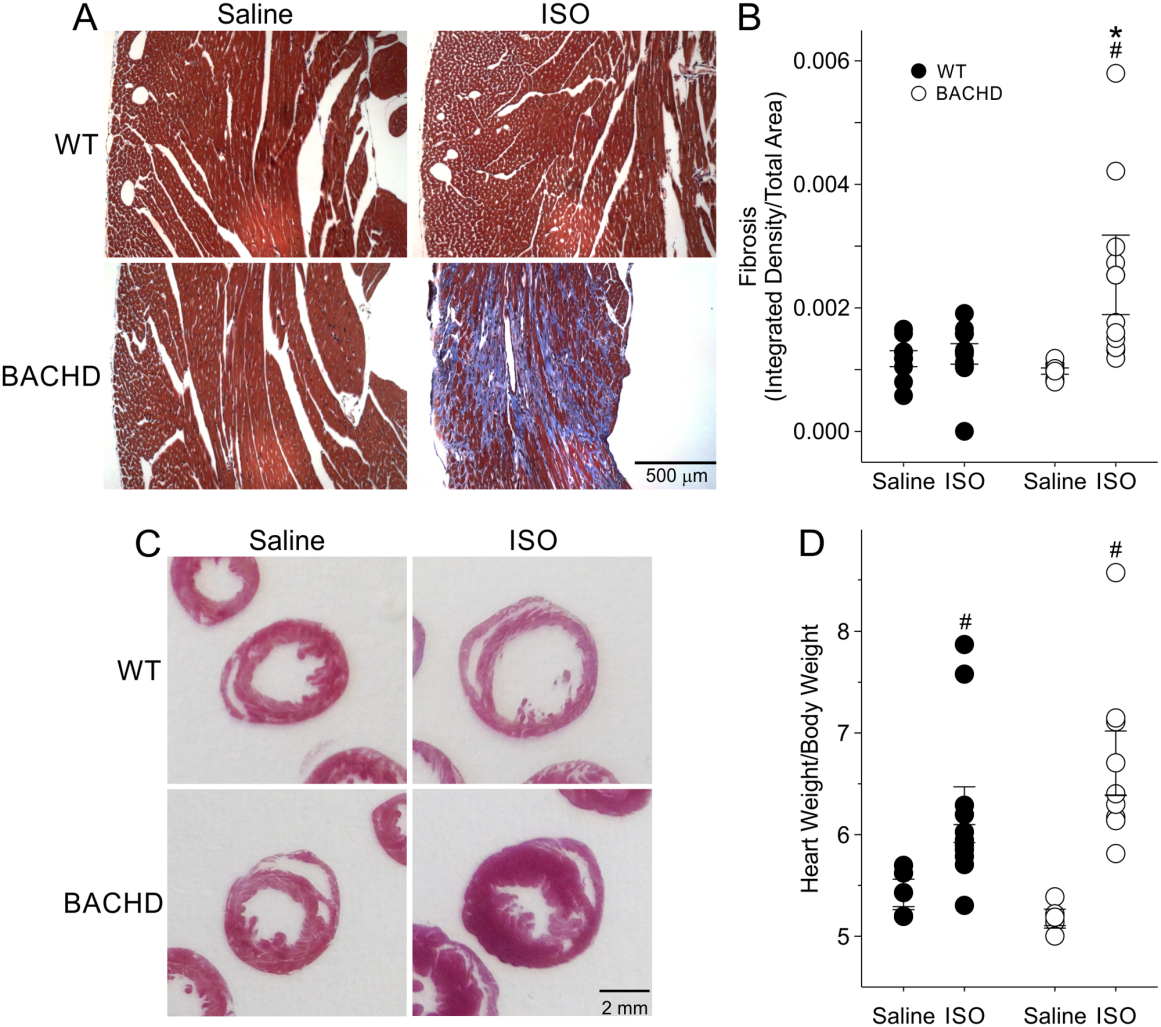
Histological analysis finds evidence of increased fibrosis in the BACHD hearts in response to the adrenoreceptor challenge. Examples of Masson’s Trichrome (A) stained heart sections from WT and BACHD mice treated with saline or ISO. Quantification of fibrosis by measurement of the optical density of blue staining confirmed increased fibrosis in the ISO-treated BACHD animals (B). Comparison of heart dimensions from H & E sections (C) and morphological measurements of WT and BACHD (D) mice indicated that the heart weight/body weight ratio was higher in both groups following ISO treatment. However, there were no significant differences between the genotypes. * P < 0.05 between genotypes; # *P*<0.05 vs. saline within a genotype.

Following the 3 months of chronic ISO treatment, we sacrificed the mice (6mo) and processed their hearts for Masson’s Trichrome to visualize fibrotic tissue or H&E staining. Masson’s trichrome staining of WT and BACHD hearts treated with saline did not reveal any positive fibrotic staining (Fig 4A). In WT mice, the ISO treatment caused positive focal lesions in 1 heart; however, 6 of 8 BACHD mice had positive fibrotic staining. Optical density measures of fibrotic staining revealed a significant increase in the fibrotic regions in ISO-treated BACHD mice compared to saline-treated BACHD mice (Fig 4B; Table 4), as well as compared to ISO-treated WT.

**Table 4:** Histological measurements of WT and BACHD hearts following isoproterenol challenge.

Histological Measurements	WT (*n* = 10)	BACHD (*n* = 8)	*t*-test
**HW/BW**	6.2 ± 0.3	6.7 ± 0.3	*T* = -1.22, *P* = 0.24
**HW/TL**	10.4 ± 0.3	11.9 ± 0.9	*T* = -1.79, *P* = 0.09
**HW (mg)**	182.0 ± 5.3	210.7 ± 16.6	*T* = -1.80, *P* = 0.09
**BW (g)**	29.7 ± 1.0	31.2 ± 1.3	*T* = -0.96, *P* = 0.35
**TL (mm)**	17.5 ± 0.1	17.7 ± 0.1	*T* = -1.12, *P* = 0.28
**Fibrosis (integrated density/area)**	12.5 ± 1.7	22.8 ± 5.2*	*T* = 1.72, *P* = 0.03

* *P*<0.05 significant difference between genotypes under the same condition.

Morphometric measurements detected heavier body weights (BW) in BACHD mice treated with saline compared to WTs (WT: 29.4 ± 1.4 g; BACHD: 34.4 ± 1.4 g; P<0.05). ISO treatment eliminated the difference in BW between genotypes (Table 4). HW was also higher in BACHD mice as compared to WT, and ISO treatment elicited an increase in the weight of the heart of both WT and BACHD mice, although larger in the BACHD. HW/BW (Fig 4D) and HW/TL ratios were not different between saline treated WT and BACHD animals (Table 4). Following ISO treatment, HW/BW was higher in ISO treated BACHD mice compared to saline treated BACHD animals (Fig 4D), but was not different when compared to ISO treated WTs. (Fig 4D, Table 4).

In line with the results in Table 1, we found that young WT and BACHD animals exhibited differences in heart dimensions at 6 mo, providing further evidence for the early structural and functional anomalies reported above. In BACHD mice, both the Lv diameter and area were significantly smaller than in WT, while exhibiting thicker Lv and septal walls (Table 5). ISO treatment increased the Lv diameters and areas in both genotypes compared to saline-treated animals, with a greater effect in the mutant hearts (Table 5). The septal and Lv wall thickness remained significantly thicker in BACHD animals (Fig 4C). No differences were seen in the right ventricle (data not shown). Notably, ISO treatment elicited significant changes in the cross sectional area of BACHD cardiomyocites (139.9 ±16.1 μm^2^, n = 5, P<0.05) as compared to WT (93.0.1 ± 14.4 μm^2^, n = 4).

**Table 5:** Histological measurements in WT and BACHD mice (6 mo) following 12 wks of isoproterenol challenge.

Histological Measurements	WT		BACHD		Two-way Repeated Measures ANOVA		
Drug	Saline (*n* = 4)	ISO (*n* = 5)	Saline (*n* = 4)	ISO (*n* = 5)	*Genotype*	*Drug*	*Interaction*
**Lv Diameter (mm)**	3.4 ± 0.2	4.1 ± 0.1*	**2.2 ± 0.2**‡	3.5 ± 0.3†	*F*_*1*_ = 13.1, *P<*0.001	*F*_*1*_ = 16.7, *P<*0.001	*F*_*1*_ = 1.26, *P* = 0.269
**Lv Area (mm**^**2**^**)**	7.12 ± 0.94	10.84 ± 0.59*	**3.21 ± 0.48**‡	8.40 ± 1.65†	*F*_*1*_ = 8.49, *P* = 0.006	*F*_*1*_ = 16.7, *P<*0.001	*F*_*1*_ = 0.46, *P* = 0.503
**Lv Thickness (mm)**	0.79 ± 0.09	0.67 ± 0.04	**1.33 ± 0.11**‡	**1.13 ± 0.17**‡	*F*_*1*_ = 18.1, *P<*0.001	*F*_*1*_ = 1.96, *P* = 0.171	*F*_*1*_ = 0.12, *P* = 0.733
**Septal Thickness (mm)**	0.70 ± 0.08	0.68 ± 0.02	**1.26 ± 0.08**‡	**1.06 ± 0.15**‡	*F*_*1*_ = 21.6, *P<*0.001	*F*_*1*_ = 1.16, *P* = 0.289	*F*_*1*_ = 0.76, *P* = 0.380
**Rv area (mm**^**2**^**)**	3.8 ± 0.5	3.1 ± 0.3	5.0 ± 0.6	3.7 ± 0.6	*F*_*1*_ = 3.47, *P* = 0.083	*F*_*1*_ = 3.58, *P* = 0.079	*F*_*1*_ = 0.28, *P* = 0.603
**Rv thickness (mm)**	0.39 ± 0.02	0.42 ± 0.04	0.47 ± 0.05	0.41 ± 0.03	*F*_*1*_ = 1.19, *P* = 0.284	*F*_*1*_ = 0.20, *P* = 0.657	*F*_*1*_ = 1.35, *P* = 0.255

* *P*<0.05 within WT, significant difference vs saline,

^†^ *P*<0.05 within HD, significant difference vs saline,

^‡^ *P*<0.05 significant difference between genotypes under the same condition.

Rv = Right ventricle.

In summary, histological examination suggests that both WT and BACHD hearts responded adaptively to ISO treatment. BACHD hearts exhibit susceptibility to developing fibrotic lesions when subject to cardiovascular challenge. Again, the data indicates that the BACHD mice develop structural abnormalities early in the disease progression and life-long hypertrophy.

### Microarray Analysis Indicates Gene Expression Changes in Apoptotic, Proliferation, Metabolism, and Immune Function in the Hearts of Young BACHD Mice

**Fig 5:**
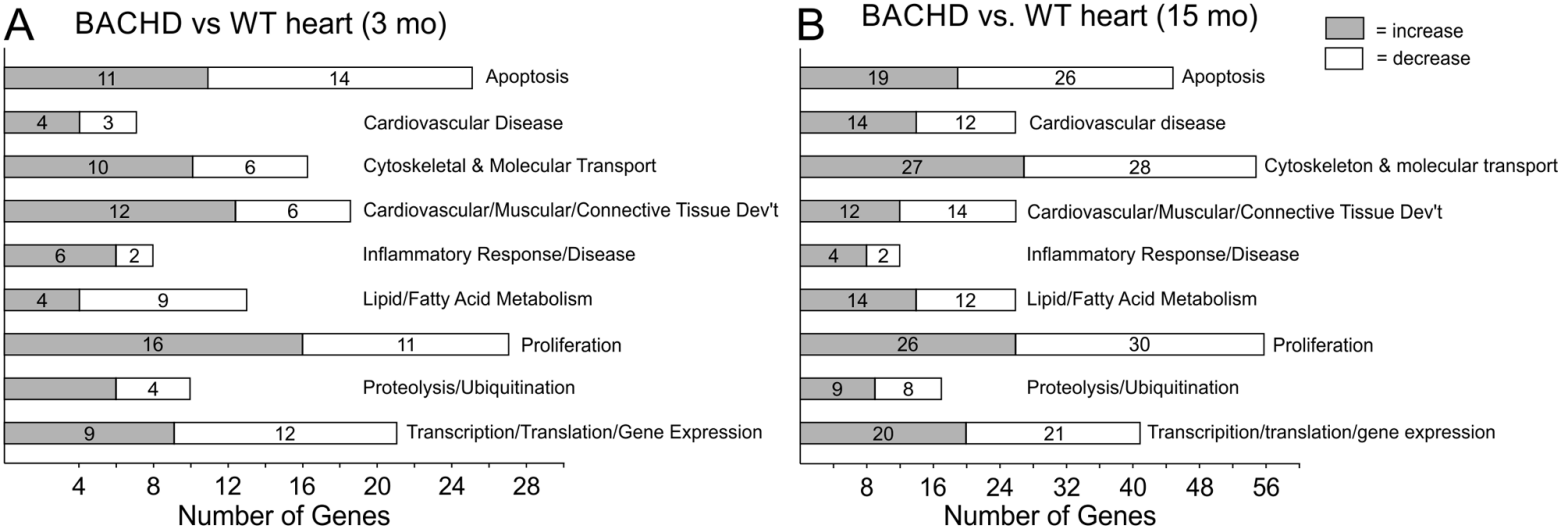
Network analysis of biologically relevant pathways altered in the heart of BACHD mice vs WT as measured by microarrays. Most altered gene networks in the comparison between young (3 mo) WT and BACHD hearts (A) and in the comparison between aged (15 mo) WT and aged BACHD (B). Grey boxes indicate that gene expression is increased in BACHD mice, while white boxes indicate decreased expression.

In order to begin to identify pathways that may be responsible for the decreased cardiovascular function in the BACHD animals, we assessed gene expression in the ventricles of young (3 mo) and older (15 mo) BACHD and WT mice using microarrays. Early in the disease progression, a total of 105 genes (50 upregulated and 55 downregulated) were moderately but significantly changed. The top 10 genes whose expression varied between the two genotypes at each age are shown in Table 5. Biologically significant pathways with altered gene expression in the ventricles of the BACHD mice included metabolic (monosaccharide and fatty acid), cytoskeletal organization, immune dysfunction and apoptosis (Fig 5A and 5B). A closer look at the expression pattern of such genes suggested that a majority of the changes in expression appears to promote apoptosis in the hearts of young BACHD mice. In fact, genes known to inhibit apoptosis were down-regulated (*Dpp7*, *Yap1*, *Mns221*) while genes that promote apoptosis were up-regulated (*Naa35*, Irf5, *Sgk3*, *Rbm3*, *Bcl212*, *Ncoa3*, *Lcmt1*, *Fanc1*, *Rbpj*, *Cd22*, *Apex1*, *Rpgrip1*). We also found significant changes in the expression of a number of genes implicated in cardiovascular disease at 15mo (Fig 5; Table 6). At this age, a total of 254 genes (149 up-regulated and 105 down-regulated) were significantly changed between the genotypes. Pathway analysis using IPA program indicates that the largest changes occurred in the cardiovascular disease, cellular metabolism, gene expression, molecular transport and proliferation clusters (Fig 5B). It is worth emphasizing that we compared the HD mutant to age-matched control tissue, hence aging alone was a major regulator of gene expression patterns. A comparison between young and old WT tissue indicates 806 genes whose expression was significantly changed with age.

**Table 6:** List of top 10 genes up-regulated and down-regulated in BACHD ventricles compared to WT at the same age (n = 4 per group).

Probe	Accession	Full Name	Symbol	Fold Change	*P* Value	Function
**Young Hearts, Down-regulated**						
ILMN_2775202	NM_027491	Ras-related GTP Binding D	Rragd	-0.348	4.00E-05	Nucleotide Binding, GTP Binding
ILMN_2835723	NM_027288.2	Mannosidase, Beta A, Lysosomal	Manba	-0.324	1.00E-04	Glycoprotein Catabolic Process
ILMN_2698107	NM_010120.3	Eukaryotic Translation Initiation Factor 1A	Eif1a	-0.205	0.00039	Protein Translation
ILMN_2925281	NM_008991.2	ATP-binding Cassette, Sub-family D	Abcd3	-0.3	0.00042	Nuclear Receptors in Lipid Metabolism and Toxicity
ILMN_2775600	NM_198619.1	Unknown	MGC67181	-0.301	0.00059	Regulation of Transcription
ILMN_3092415	NM_153198.1	High Mobility Group Box Transcription Factor 1	Hbp1	-0.392	0.00071	Wnt Receptor Signaling Pathway, Regulation of Transcription
ILMN_3006930	NM_027409.1	Motile Sperm Domain Containing 1	Mospd1	-0.215	0.00072	Integral to Membrane, Intrinsic to Membrane
ILMN_2661733	NM_008212.1	L-3-Hydroxyacyl-Coenzyme A Dehydrogenase, Short Chain	Hadhsc	-0.378	0.00105	Fatty Acid Elongation in Mitochondria, Fatty Acid Metabolism
ILMN_1243283	NM_010771.3	Matrin 3	Matr3	-0.303	0.00107	RNA Polymerase III Transcription
ILMN_2512043	NM_009454.2	Ubiquitin-Conjugating Enzyme E2E 3	Ube2e3	-0.306	0.00117	Ubiquitin Mediated Proteolysis
**Young Hearts, Up-regulated**						
ILMN_1250767	NM_025482.2	Tumor Protein D52-like 2	Tpd52l2	0.254	2.00E-05	Tumor Protein D52
ILMN_1234565	NM_011030.1	Procollagen-proline, 2-Oxoglutarate 4-Dioxygenase	P4ha1	0.407	0.00026	Arginine and Proline Metabolism
ILMN_1255832	NM_153134.2	Immunity-related GTPase Family, Q	Irgq	0.229	0.00029	Immunity-related GTPase Family, Q
ILMN_2749737	NM_031843.2	Dipeptidylpeptidase 7	Dpp7	0.207	0.00038	Proteolysis
ILMN_2486906	NM_016873.1	WNT1 Inducible Signaling Pathway Protein 2	Wisp2	0.344	0.00043	Regulation of Cell Growth, Cell Adhesion
ILMN_1258691	NM_133226.1	PDZ Domain Containing 2	Pdzk2	0.258	5.00E-04	Guanylate Cyclase Inhibitor Activity
ILMN_2930933	NM_008717.1	Zinc Finger, Matrin-like	Zfml	0.299	6.00E-04	DNA Binding, RNA Binding; Ion, Metal, Cation Binding
ILMN_1233813	NM_009280	Synovial Sarcoma Translocation, Chromosome 18	Ss18	0.215	0.00062	Microtubule Cytoskeleton Organization
ILMN_2683414	NM_033568.1	ESCRT-II Complex Subunit	D11Moh34	0.245	0.00066	Endocytosis
ILMN_2615547	NM_030262	O-Fucosyltransferase 2	Pofut2	0.232	0.00079	Monosaccharide Metabolic Process
**Aged Hearts, Down-regulated**						
ILMN_2625893	NM_053200.1	Carboxylesterase 3	Ces3	-0.379	0	Fatty Acid/Lipid Metabolism
ILMN_2925281	NM_008991.2	ATP-binding Cassette, Sub-family D (ALD), Member 3	Abcd3	-0.445	1.00E-05	Peroxisome
ILMN_2834915	NM_011396.2	Solute Carrier Family 22 (organic cation/carnitine transporter), Member 5	Slc22a5	-0.415	1.00E-05	Molecular Transport
ILMN_2835723	NM_027288.2	Mannosidase, Beta A, Lysosomal	Manba	-0.345	5.00E-05	Glycoprotein Catabolic Process
ILMN_1234702	NM_173866.1	Glutamic Pyruvate Transaminase 2	Gpt2	-0.371	8.00E-05	Mitochrondrion, Transferase Activity
ILMN_3114124	NM_133943.2	Hydroxy-delta-5-steroid Dehydrogenase, 3 Beta- and Steroid delta-isomerase 7	Hsd3b7	-0.402	9.00E-05	Lipid Biosynthetic Process
ILMN_2966162	NM_178936.3	Transmembrane Protein 56	Tmem56	-0.319	0.00011	Transmembrane
ILMN_1240332	NM_146073.2	Zinc Finger, DHHC Domain Containing 14	Zdhhc14	-0.343	0.00012	Ion Binding
ILMN_2794051	NM_172734.2	Serine/Threonine Kinase 38 Like	Stk38l	-0.295	0.00014	Actin-Cytoskeleton
ILMN_2973925	NM_026398.2	Processing of Precursor 5	Pop5	-0.292	0.00015	RNA Metabolic Process
**Aged Hearts, Up-regulated**						
ILMN_2718416	NM_028659.1	Eukaryotic Translation Initiation Factor 3, Subunit K	Eif3k	0.339	2.00E-05	Protein Synthesis
ILMN_2775202	NM_027491	Ras-related GTP Binding D	Rragd	0.358	3.00E-05	Nucleotide Binding
ILMN_1239181	NM_026393.1	NmrA-like Family Domain Containing 1	Nmral1	0.378	7.00E-05	Oxidation Reduction, Transcripiton Regulator Activity
ILMN_2650739	NM_146011.1	Rho GTPase Activating Protein 9	Arhgap9	0.405	1.00E-04	GTPase Activator Activity
ILMN_1215768	NM_024182.2	RIO Kinase 3	Riok3	0.266	0.00012	Phosphorus Metabolic Process
ILMN_2647048	NM_023721.1	ATPase, H+ Transporting, Lysosomal V1 Subunit D	Atp6v1d	0.333	0.00015	Purine Nucleotide Metabolic Process
ILMN_2929572	NM_201362.1	Coiled-Coil Domain Containing 68	Ccdc68	0.282	0.00016	Coiled-coil Domain Containing 68
ILMN_2986315	NM_019580.3	Membrane Interacting Protein of RGS16	Mir16	0.205	0.00019	Glycerol Metabolic Process
ILMN_2879600	NM_008983.1	Protein Tyrosine Phosphatase, Receptor Type, K	Ptprk	0.234	3.00E-04	Protein Dephosphorylation
ILMN_3161547	NM_008725.1	Natriuretic Peptide Type A	Nppa	1.316	3.00E-04	Cardiovascular/ Muscular Development

### PCR Measurements Found Gene Expression Changes in the Hearts of Young BACHD Mice

**Fig 6:**
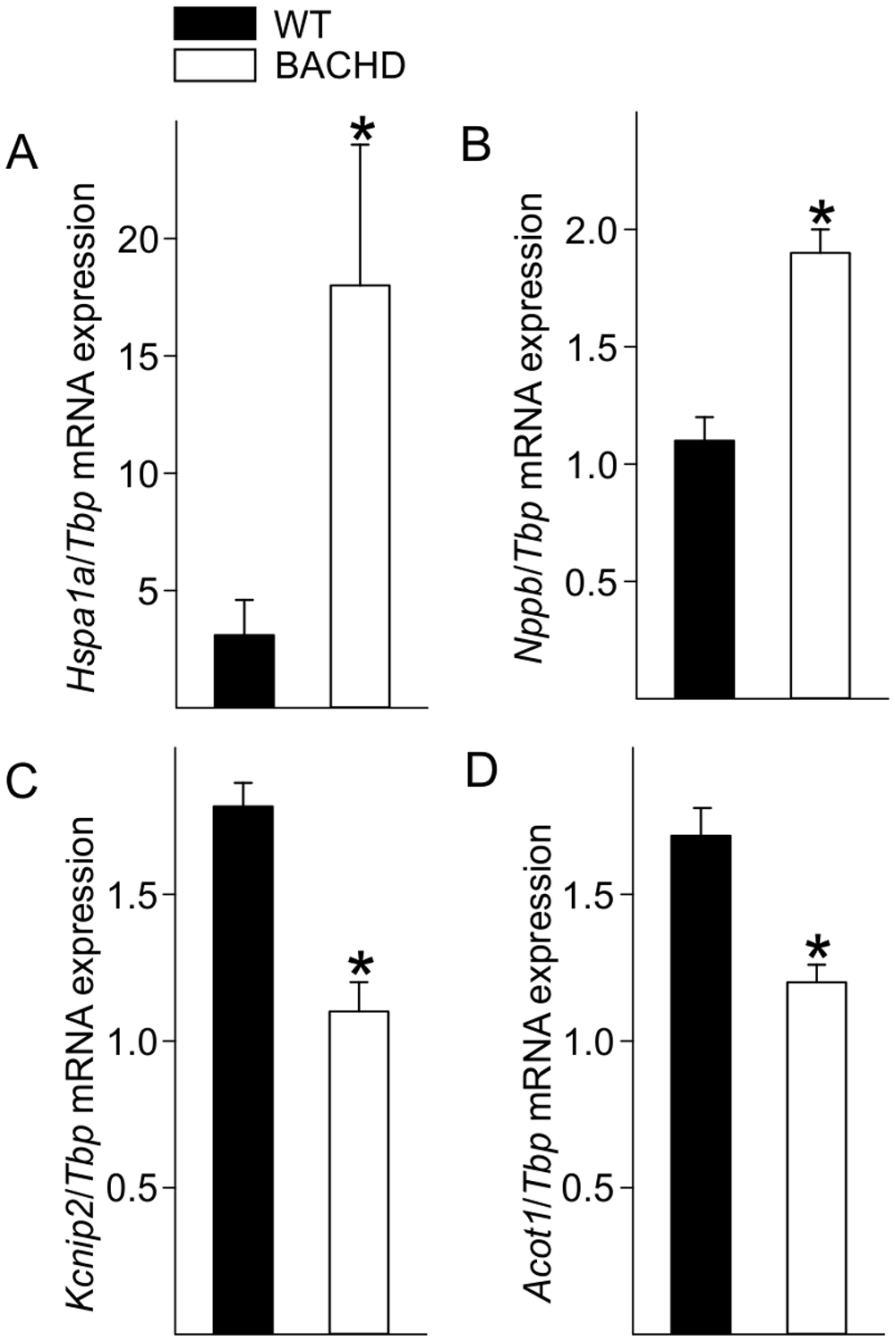
Microarray results were confirmed by probing expression of 4 genes using quantitative real-time PCR from ventricles from mice at 3 mo of age. *Hspa1a* (A) and *Nppb* (B) are both involved in stress and pathological responses and were increased in the BACHD mice. Expression of *Kcnip2* (C), a voltage-gated potassium channel interacting protein responsive to changes in calcium, and *Acot1* (D) an enzyme involved in fatty acid metabolism were decreased in BACHD ventricles. Data are reported as ratios of the target gene expression to *Tbp*, and are shown as the mean ± SEM, * *P*<0.05 for genotypic differences (n = 4 per group).

We further examined transcriptional differences by measuring the expression of 4 genes in the ventricles of each genotype using using quantitative real-time PCR (Fig 6A–6D). We chose 2 genes (*Hspa1a*, *Nppb*) that were shown to be up-regulated by the microarray analysis and 2 genes (*Kcnip2*, *Acot1*) whose expression was shown to be down-regulated. All of the genes have been previously shown to be involved in cardiomyopathy. In each case, the PCR measurements were in agreement with the microarray results (Fig 6A–6D).

### Altered Inflammation in Young BACHD Mice

**Fig 7:**
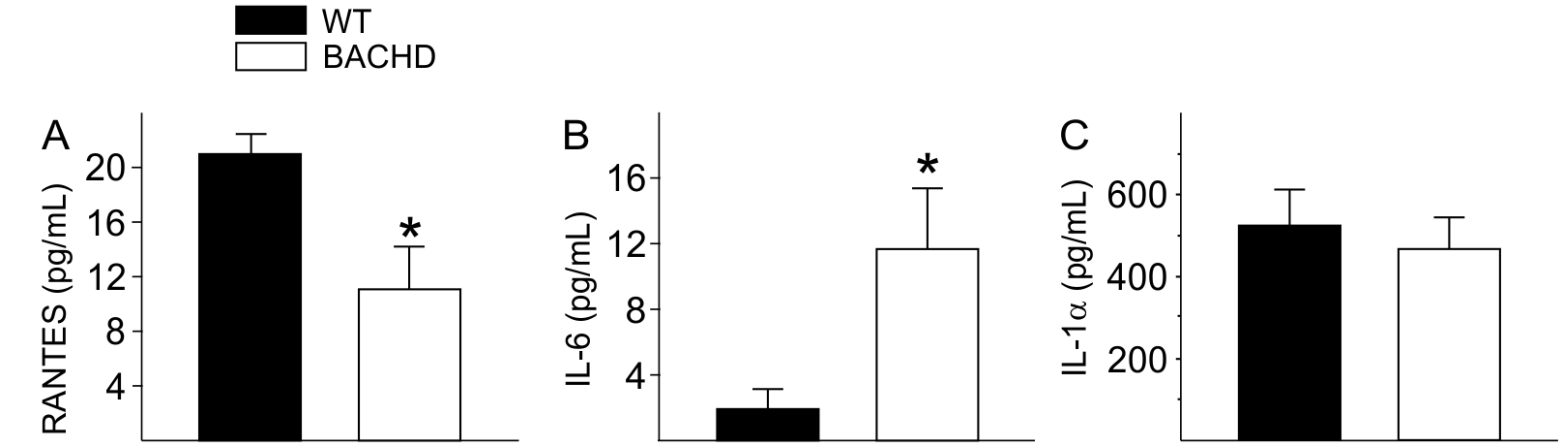
Altered inflammatory markers in young BACHD mice. Levels of chemokines and pro-inflammatory cytokines were measured in the serum of young (3 mo) BACHD and WT mice using a multiplex assay. Significant changes in RANTES (A) and IL-6 (B) levels were found in serum of young WT and BACHD mice. IL-1α (C) levels were not different. Data are shown as the mean ± SEM, * *P*<0.05 for genotypic differences (n = 8 per group).

The microarray results suggested that BACHD mice have altered inflammatory response with up-regulated genes such as *Tnfrsf25* and *Adam9*. In order to confirm this finding, we measured the levels of immune factors in the serum of young (2–3 mo) BACHD and WT mice using multiplex assay. At baseline, without immune challenge, there was a significant decrease in the levels of the CC chemokine RANTES (Regulated on Activation, Normal T Cell Expressed and Secreted) as well as a significant increase in the levels of the pro-inflammatory cytokine interleukin (IL)-6 (Fig 7A and 7B). We found no changes in the cytokine IL-1α levels between genotypes (Fig 7C), and an absence of signal for several others (TNF-α, IL-10, IL-12, IL-1β, IL-2 and IFN-γ) and the chemokines MIP1α (Macrophage inflammatory protein 1 alpha) and MCP-1 (Monocyte Chemoattractant Protein-1 (MCP-1) were also undetectable. The changes in levels of IL-6 and RANTES are consistent with microarray results that suggest an altered inflammatory state in young BACHD mice.

### Increased Caspase 3 Levels in Aged BACHD Mice

**Fig 8:**
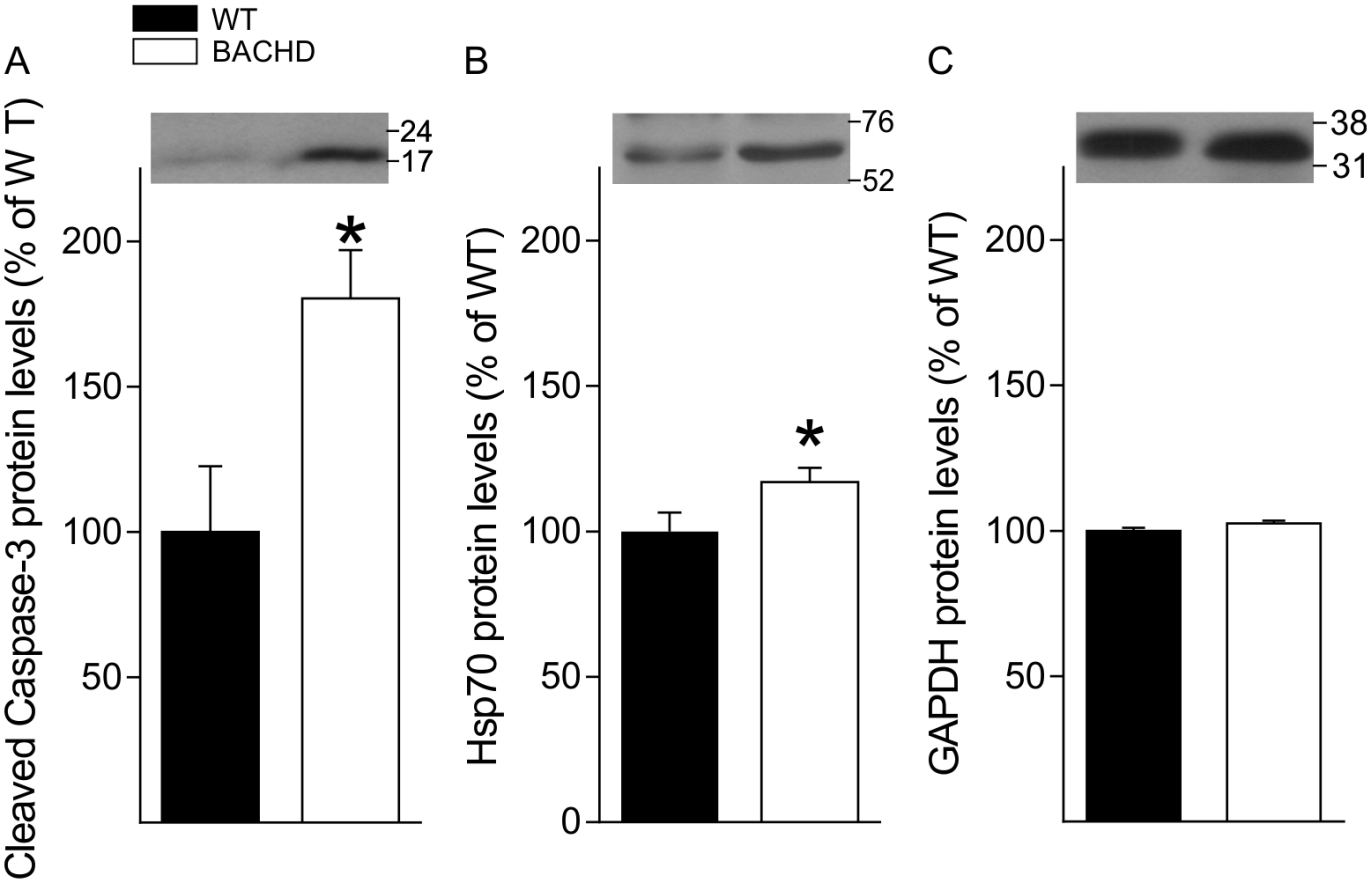
Apoptotic and stress markers are increased in the aged BACHD heart. A significant increase in Cleaved Caspase 3 (A) and Hsp-70 (B) protein levels was found in whole tissue lysate of the heart of aged BACHD mice. Values derived from the densitometric analysis were corrected for the background, normalized to GAPDH and are shown as a percentage of the value for the WT mice. (C) Protein levels of GAPDH did not differ between WT and BACHD. Insert shows representative immunoblots. Data are shown as the mean ± SEM, * *P*<0.05 for genotypic differences (n = 4 per group).

One of the most populated clusters of genes altered in the hearts of both young and old BACHD mice was the one involved in apoptosis. Therefore, we measured the protein levels of the cell death effector caspase-3, in whole tissue lysate from aged BACHD and WT hearts. The levels of the activated form of this protein were significantly increased (80%, Fig 8A) in the BACHD mice as compared to WT. We also found a smaller (17%, Fig 8B) but significant increase in expression of HSP70. These changes in protein are consistent with the microarray analysis in showing increases in genes involved in cellular stress and apoptosis in the BACHD heart.

## Discussion

The echocardiographic measurements performed on BACHD mice showed changes in heart structure and function at an early age (Fig 1). At this time point (3 mo), motor deficits [24] and circadian rhythm dysfunction [15] are just beginning thus the cardiovascular deficits are occurring very early in the disease progression in this model. Though the slope of the changes were similar between aging WT and BACHD animals, the ejection fraction (EF) ratio of 15 mo old BACHD mice were close to or below 55, which is the threshold between normal heart function and failure. We suspect that the function would continue to weaken with age, which poses a challenge for the cardiovascular system, leaving BACHD mice more susceptible to serious cardiac events. The presence of cardiac fibrosis in 15 mo old BACHD mice (Fig 2) also provides evidence for cardiac stress that feeds forward to further impair heart function. WT mice show minimal fibrotic lesions at this age. The focal fibrotic lesions in the BACHD would be expected to alter wall motion synchrony and could explain the reduced EF. Evidence for fibrosis has also been found in the R6/2 line [23]. The presence of fibrosis could also increase the likelihood of arrhythmias [29] although we did not observe any in our recordings. Though the incidence of arrhythmias has not yet been examined in HD patients, other lines of evidence suggest increased susceptibility including altered ANS input to the heart [11, 30]. ANS dysfunction is consistent among a number of HD animal models including the R6/1 [17] and the R6/2 models [18]. In the BACHD model, we have found significant alterations in the baroreceptor reflex and a decline in heart-rate variability that indicate dysfunctional ANS outflow [16]. With this work, we have provided evidence that BACHD line recapitulates cardiovascular dysfunction seen in HD patients as well as established an age-dependent progression by which we can judge the impact of therapeutic interventions.

Despite the differences in structural and functional measurements, the hearts of BACHD mice appear to, at least in the short-term, adapt normally to increased cardiovascular challenge. Isoproterenol is a medication used for the treatment of bradycardia (slow heart rate) and heart block. By activating beta-adrenergic receptors on the heart, it increases heart rate and mimics the action of the sympathetic nervous system. The response to isoproterenol, as measured by echocardiography, was not different between WT and BACHD mice (Fig 3). The chronic β-adrenergic stimulation even caused the mutant mice to lose weight and to move into age-appropriate body weights (Table 4). However, upon histological assessment, this adaptation came at a cost i.e. the development of fibrosis in the BACHD hearts (Fig 4). WT mice subject to similar treatment did not produce cardiac fibrosis, indicating that the cardiac state in BACHD mice is susceptible to aberrant changes and scarring when presented with this challenge.

Finally, to better define the transcriptional state of the BACHD hearts, we used microarrays to compare gene expression of young (3 mo) and older (15 mo) hearts to determine if we could detect an HD “signature” in the ventricles (Fig 5; Table 6). The results showed alteration in pathways and processes that are broadly consistent with the results of profiling studies of other tissues [31–33]. For example, genes with altered expression in the heart of BACHD mice (*Hspa1a*, *P4ha1*, *Tpd52l2*, *Basp1*, *Hadh*, *Baiap2*, *Fgf13*, Zfp932, *Hbp1)* were previously characterized to play an important role in HD pathology in other tissues. Altered pathways in the heart that were consistent with the HD signature include protein processing and ubiquitination, metabolic processes, transcriptional activities and cytoskeletal functions. Importantly, we also detected alterations in cardiac relevant processes including gene networks associated with cardiovascular development and disease (Fig 5; Table 7). Among other findings, our microarray and PCR (Fig 6) results indicated that *Kcnip2* levels were significantly decreased at 3 mo of age. *Kcnip2* is a voltage-gated potassium channel cofactor known to alter electrical properties of the heart and altered expression levels can increase susceptibility to arrhythmias [34, 35].

**Table 7:** List of cardiovascular disease related genes altered in BACHD ventricles compared to WT at the same age.

Probe	Accession	Enzyme name	Symbol	Fold Change	*P* value
**Young Hearts (3 mo)**					
ILMN_2689473	NM_007381.2	Acyl-Coenzyme A Dehydrogenase, Long-Chain	Acadl	-0.354	0.00229
ILMN_2659063	NM_013457	Adducin 1	Add1	-0.159	0.0032
ILMN_2726837	NM_008726.2	Natriuretic Peptide Precursor Type B	Nppb	0.466	0.00083
ILMN_1248293	NM_009534.1	Yes-associated Protein 1	Yap1	0.29	0.00236
ILMN_2735350	NM_011819.1	Growth Differentiation Factor 15	Gdf15	0.376	0.00337
ILMN_2726837	NM_008726.2	Phosphodiesterase 8A	Pde8a	0.24	0.00433
**Aged Hearts (15 mo)**					
ILMN_3008858	NM_009982.2	Cathepsin C	Ctsc	-0.263	0.00055
ILMN_2721439	NM_007792.2	Cysteine and Glycine-rich Protein 2	Csrp2	-0.335	7.00E-04
ILMN_2885532	NM_009949.1	Carnitine Palmitoyl Transferase 2	Cpt2	-0.311	0.0015
ILMN_2886828	NM_011243.1	Retinoic Acid Receptor, Beta	Rarb	-0.371	0.00194
ILMN_2727432	NM_138672.1	Stabilin 1	Stab1	-0.166	0.0021
ILMN_1229161	NM_007932	Endoglin	Eng	-0.356	0.00258
ILMN_2678336	NM_007795.1	Cardiotrophin 1	Ctf1	-0.185	0.00316
ILMN_2933647	NM_019802.1	Gamma-glutamyl Carboxylase	Ggcx	-0.252	0.00377
ILMN_2724942	NM_008968.2	Prostaglandin I2 (prostacyclin) Synthase	Ptgis	-0.27	0.00382
ILMN_1223738	NM_011034.2	Peroxiredoxin 1	Prdx1	-0.168	0.00383
ILMN_1232231	NM_028500	Calreticulin 3	Calr3	-0.297	0.00446
ILMN_2613904	NM_024441.1	Heat Shock Protein 2	Hspb2	-0.241	0.00456
ILMN_2945491	NM_080728.2	Myosin, Heavy Polypeptide 7	Myh7	1.756	0.00046
ILMN_2453051	NM_023500	X-linked Kx Blood Group	Xkh	0.153	0.00062
ILMN_2716567	NM_009761.2	BCL2/adenovirus E1B Interacting Protein 3-like	Bnip3l	0.391	0.00071
ILMN_1224768	NM_133838	EH-domain Containing 4 (Ehd4)	Ehd4	0.336	0.00101
ILMN_1235481	NM_021880.1	Protein Kinase, cAMP Dependent Regulatory, Type I, Alpha	Prkar1a	0.239	0.00126
ILMN_2667369	NM_022305.2	UDP-Gal:betaGlcNAc Beta 1,4- Galactosyl Transferase, Polypeptide 1	B4galt1	0.186	0.00159
ILMN_2677634	NM_172621.1	Chloride Intracellular Channel 5	Clic5	0.324	0.00311
ILMN_2711948	NM_011404.2	Solute Carrier Family 7 (Amino Acid Transporter Light Chain, L System), Member 5	Slc7a5	0.261	0.00326
ILMN_2594450	NM_008960	Phosphatase and Tensin Homolog	Pten	0.188	0.00329
ILMN_1255207	NM_008803.1	Phosphodiesterase 8A	Pde8a	0.249	0.00335
ILMN_2625451	NM_013468.2	Ankyrin Repeat Domain 1	Ankrd1	0.559	0.00391
ILMN_2689136	NM_008653.1	Myosin Binding Protein C, Cardiac	Mybpc3	0.278	0.00469

In the BACHD young heart, microarray analysis reveals increased expression of *Nppb*, also known as brain natriuretic peptide. Studies have suggested that increased level of *Nppb* is related to higher risk of myocardial fibrosis and ventricular geometry [36, 37]. In addition, the altered expression of genes encoding heat-shock proteins, family A and B (*Hspa1a*, *Hspb2*) and lysosomal enzymes (*Manba*) suggests protein misfolding and cellular stress in young hearts. Recent evidence supports a role for Hsp70 in heart hypertrophy [38], coherently, we found elevated levels of of this protein in aged mutant hearts (Fig 8B). We found that the total number of gene changed is higher in the aged hearts. In fact, there are 105 genes changed in the young comparison while 254 genes changed in the old comparison. It is worth emphasizing that age might dampen the HD signature in the older hearts (15mo of age). Yet, we found that *Acot1* is down-regulated in the old heart of BACHD mice. A decrease in *Acot1* expression is associated with cardiac myopathy and damage [39]. Therefore, our microarray supports the assertion that the hearts of BACHD mice have higher risk of fibrosis and arrhythmia since young age, and it contributes to increased susceptibility to cardiac dysfunction with age.

The microarray analysis found that the transcription of a number of genes involved in the inflammatory response were altered, which led us to measure cytokine levels in the serum of BACHD mice. Parallel to microarray results, we found increased IL-6 levels as well as decreased RANTES in the serum of non-stimulated BACHD mice early in the disease progression (Fig 7). IL-6 is a pro-inflammatory cytokine, which has been involved in cardiac remodeling by inducing Lv hypertrophy and increasing collagen deposits [40] and therefore could contribute to the cardiac pathology in BACHD mice. Increased levels of IL-6 have been detected in the plasma and brain tissue of HD patients [41, 42], with indications that the dysregulation begins during pre-symptomatic stages of the disease [43]. Consistent with our results, RANTES/CCL5 secretion from astrocytes of R6/2 is also reduced. The effects of altered RANTES levels in serum on cardiovascular health continue to be explored, but there is evidence that reduced RANTES is associated with myocardial infarction, atherosclerosis and cardiac mortality [44] and therefore could also contribute to the cardiac pathology in HD.

Compared with age-matched hearts of WT controls, our microarray analysis reveals down-regulation of genes that suppress apoptosis pathway and up-regulation of genes that promote apoptosis pathway in both young and aged hearts of the BACHD mice (Table 8). The findings from the young heart were intriguing. For example, as early as 3 mo of age, the expression of *Bcl2l12* is down-regulated while the expression of *Tnfrsf25* and *Irf5* are up-regulated in BACHD hearts. Bcl2l12 is an anti-apoptotic factor that acts as an inhibitor of caspases 3 and 7 in the cytoplasm. In the nucleus, it binds to the tumor suppressor p53, preventing its association with target genes. Overexpression of this gene has been detected in a number of different cancers. On the other hand, *Tnfrsf25* is a member of the TNF-receptor superfamily. This receptor has been shown to stimulate NF-kappa B activity and regulate cell apoptosis. Therefore, Tnfrsf25 has been shown to involve TNF-mediated signal pathway and promote apoptosis process. Similarly, *Irf5* is a member of the interferon regulatory factor family, a group of transcription factors with diverse roles, including virus-mediated activation of interferon, and modulation of cell growth, differentiation, apoptosis, and immune system activity. In short, our microarray findings support that promoted apoptosis process of BACHD young heart may increase the susceptibility of fibrosis in BACHD mice. Western blot data confirmed that the levels of the apoptotic marker Caspase 3 were indeed elevated in the BACHD heart (Fig 8A).

**Table 8:** List of apoptosis related genes altered in BACHD ventricles compared to WT at the same age.

Probe	Accession	Enzyme name	Symbol	Fold Change	*P* value
**Young Hearts (3 mo)**					
ILMN_1225608	NM_008679	Nuclear Receptor Coactivator 3	Ncoa3	-0.179	0.00107
ILMN_3079866	NM_009845.1	Cluster of Differentiation-22	Cd22	-0.155	0.00138
ILMN_3035162	NM_133220.2	Serum/Glucocorticoid Regulated Kinase 3	Sgk3	-0.178	0.00145
ILMN_2943477	NM_025304.3	Leucine Carboxyl Methyl Transferase 1	Lcmt1	-0.163	0.00157
ILMN_2793522	NM_013869.3	Tumor Necrosis Factor Receptor Superfamily, Member 19	Tnfrsf19	-0.198	0.00251
ILMN_1247125	NM_025923.2	Fanconi Anemia, Complementation Group L	Fancl	-0.174	0.00262
ILMN_2776952	NM_009687	Apurinic/Apyrimidinic Endonuclease 1	Apex1	-0.145	0.00294
ILMN_2675776	NM_023879	Retinitis Pigmentosa GTPase Regulator Interacting Protein 1	Rpgrip1	-0.117	0.00406
ILMN_1236489	NM_029410.1	BCL2-like 12	Bcl2l12	-0.213	0.00435
ILMN_2866752	NM_007530.2	B-cell Receptor-associated Protein 29	Bcap29	-0.149	0.00435
ILMN_1234453	NM_016809.2	RNA Binding Motif Protein 3 (Rbm3)	Rbm3	-0.553	0.00466
ILMN_2749737	NM_031843.2	Dipeptidylpeptidase 7	Dpp7	0.207	0.00038
ILMN_2726837	NM_008726.2	Natriuretic Peptide Precursor Type B	Nppb	0.466	0.00083
ILMN_1232240	NM_026238.2	Nuclear Prelamin A Recognition Factor-like	Narfl	0.173	0.00106
ILMN_1248293	NM_009534.1	Yes-associated Protein 1	Yap1	0.29	0.00236
ILMN_2621752	NM_012057.1	Nterferon Regulatory Factor 5	Irf5	0.182	0.00261
ILMN_2829594	NM_010479.2	Heat Shock Protein 1A	Hspa1a	1.42	0.00284
ILMN_2510383	NM_033042.2	Tumor Necrosis Factor Receptor Superfamily, Member 25	Tnfrsf25	0.259	0.00307
ILMN_2735350	NM_011819.1	Growth Differentiation Factor 15	Gdf15	0.376	0.00337
ILMN_2611812	NM_027395	Membrane Attached Signal Protein 1 (Basp1)	Basp1	0.152	0.00426
**Aged Hearts (15 mo)**					
ILMN_2886828	NM_011243.1	Retinoic Acid Receptor, Beta	Rarb	-0.371	0.00194
ILMN_2968211	NM_010706.1	Lectin, Galactose Binding, Soluble 4	Lgals4	-0.366	0.00222
ILMN_1229161	NM_007932	Endoglin	Eng	-0.356	0.00258
ILMN_1235374	NM_026172.3	2,4-Dienoyl CoA Reductase 1	Decr1	-0.311	0.0029
ILMN_2657175	NM_007585.2	Annexin A2	Anxa2	-0.292	0.00482
ILMN_2623591	NM_009685.1	Amyloid Beta (A4) Precursor Protein-binding, Family B, Member 1	Apbb1	-0.283	0.0013
ILMN_2724942	NM_008968.2	Prostaglandin I2 (prostacyclin) Synthase	Ptgis	-0.27	0.00382
ILMN_2594714	NM_011809.2	E26 Avian Leukemia Oncogene 2	Ets2	-0.255	0.00291
ILMN_2613904	NM_024441.1	Heat Shock Protein 2	Hspb2	-0.241	0.00456
ILMN_2592093	NM_018854.3	Intraflagellar Transport Protein 20	Ift20	-0.235	0.00103
ILMN_2724386	NM_177613.2	Cell Division Cycle 34	Cdc34	-0.222	0.0034
ILMN_2991107	NM_029342.3	Non-Homologous End-Joining Factor 1	Nhej1	-0.218	0.00051
ILMN_2589651	NM_008486.1	Alanine Aminopeptidase	Anpep	-0.204	0.00129
ILMN_2480180	NM_016862	Vesicle Transport Through Interaction with t-SNAREs 1A	Vti1a	-0.186	0.00377
ILMN_2678336	NM_007795.1	Cardiotrophin 1	Ctf1	-0.185	0.00316
ILMN_1223738	NM_011034.2	Peroxiredoxin 1	Prdx1	-0.168	0.00383
ILMN_2726271	NM_133205.1	Arrestin 3, retinal	Arr3	-0.164	0.00463
ILMN_1228796	NM_023179.2	ATPase, H+ Transporting, Lysosomal V1 Subunit G2	Atp6v1g2	-0.162	0.00392
ILMN_2598703	NM_010509.1	Interferon (alpha and beta) Receptor 2	Ifnar2	-0.148	0.00378
ILMN_1224846	NM_153785.1	Cyclin-dependent Kinase-like 3	Cdkl3	0.138	0.00139
ILMN_2722864	NM_010875	Neural Cell Adhesion Molecule 1	Ncam1	0.148	0.00304
ILMN_1228881	NM_013523.2	Follicle Stimulating Hormone Receptor	Fshr	0.155	0.00105
ILMN_1216788	NM_172773.1	Solute Carrier Family 17	Slc17a5	0.163	0.00124
ILMN_1215951	NM_138315.1	Microtubule Associated Monoxygenase, Calponin and LIM Domain Containing 1	Mical1	0.176	0.00473
ILMN_2667369	NM_022305.2	Beta-1,4-Galactosyl Transferase 1	B4galt1	0.186	0.00159
ILMN_2594450	NM_008960	Phosphatase and Tensin Homolog	Pten	0.188	0.00329
ILMN_2680549	NM_029094.1	Phosphatidylinositol-4,5-bisphosphate 3-kinase Catalytic Subunit Beta Isoform	Pik3cb	0.197	0.00383
ILMN_3097726	NM_178846.1	Guanine Nucleotide Binding Protein-like 3	Gnl3	0.203	0.00303
ILMN_2661125	NM_019791.1	Melanoma Antigen, Family D, 1	Maged1	0.205	0.00082
ILMN_1229318	NM_172665.1	Pyruvate Dehydrogenase Kinase, Isoenzyme 1	Pdk1	0.209	0.00393
ILMN_2840091	NM_011673.2	UDP-glucose Ceramide Glucosyltransferase	Ugcg	0.215	0.00459
ILMN_1250213	NM_019464.1	SH3-domain GRB2-like B1	Sh3glb1	0.221	0.00444
ILMN_1220096	NM_028193.1	Belgischer Rundfunk 1	Brf1	0.232	0.00173
ILMN_1235481	NM_021880.1	Protein Kinase, cAMP Dependent Regulatory, Type I, Alpha	Prkar1a	0.239	0.00126
ILMN_1259127	NM_026602.1	Breast Carcinoma Amplified Sequence 2	Bcas2	0.297	0.0031
ILMN_1259174	NM_009132.1	Scinderin	Scin	0.308	0.00243
ILMN_2727503	NM_008343	Insulin-like Growth Factor Binding Protein 3	Igfbp3	0.314	0.0041
ILMN_1224768	NM_133838	EH-domain Containing 4	Ehd4	0.336	0.00101
ILMN_2676606	NM_181848.3	Optineurin	Optn	0.34	0.00175
ILMN_2434472	NM_009413.1	Tumor Protein D52-like 1	Tpd52l1	0.388	0.0024
ILMN_2716567	NM_009761.2	BCL2/Adenovirus E1B Interacting Protein 3-like	Bnip3l	0.391	0.00071
ILMN_2728729	NM_011521.1	Syndecan 4	Sdc4	0.444	0.0014
ILMN_1226016	NM_198885.2	Scleraxis	Scx	0.458	0.00035
ILMN_2625451	NM_013468.2	Ankyrin Repeat Domain 1	Ankrd1	0.559	0.00391
ILMN_3161547	NM_008725.1	Natriuretic Peptide Type A	Nppa	1.316	3.00E-04

Many studies on HD are focused on the pathology in the brain, but it is becoming clear that the heart is highly susceptible to the effects of mHTT. Like the brain, the heart is a highly metabolic organ and our microarray studies suggest alterations in metabolic pathways in the hearts of BACHD mice. Mismatches in energy supply and demand can lead to ROS generation, cardiomyocyte damage and heart failure [45]. In addition, there are indications of changes in insulin sensitivity and misregulation of plasma glucose levels in HD patients [46], contributing to energy mishandling and cardiac pathology. Lastly, the state of the immune system is closely associated with the cardiac health, and the observed imbalances in immune factors may be driving pathogenesis. With HD, there are multiple insults that may be leading to cardiac pathology and each of these factors would likely need to be addressed in order to help manage cardiovascular concerns in the HD patient population. Overall, our data suggest that monitoring of the cardiovascular system in HD patients should start at an early age in order to intervene or slow down the progression of cardiovascular disease and delay or prevent early death. It is also worth considering that a compromised cardiovascular system could contribute to a decline in nervous system performance and result in cognitive dysfunction.
